# Effects of Polyphenol Intake on Metabolic Syndrome: Current Evidences from Human Trials

**DOI:** 10.1155/2017/5812401

**Published:** 2017-08-15

**Authors:** Gemma Chiva-Blanch, Lina Badimon

**Affiliations:** ^1^Cardiovascular Science Institute (ICCC)-Biomedical Research Institute Sant Pau (IIB-Sant Pau), Hospital de la Santa Creu I Sant Pau, Barcelona, Spain; ^2^CiberCV, Institute Carlos III, Barcelona, Spain

## Abstract

Metabolic syndrome (MetS) is a cluster of cardiovascular risk factors which severely increases the risk of type II diabetes and cardiovascular disease. Several epidemiological studies have observed a negative association between polyphenol intake and MetS rates. Nevertheless, there are relatively small numbers of interventional studies evidencing this association. This review is focused on human interventional trials with polyphenols as polyphenol-rich foods and dietary patterns rich in polyphenols in patients with MetS. Current evidence suggests that polyphenol intake has the potential to alleviate MetS components by decreasing body weight, blood pressure, and blood glucose and by improving lipid metabolism. Therefore, high intake of polyphenol-rich foods such as nuts, fruits, vegetables, seasoning with aromatic plants, spices, and virgin olive oil may be the cornerstone of a healthy diet preventing the development and progression of MetS, although there is no polyphenol or polyphenol-rich food able to influence all MetS features. However, inconsistent results have been found in different trials, and more long-term randomized trials are warranted to develop public health strategies to decrease MetS rates.

## 1. Introduction

Metabolic syndrome (MetS) is a cluster of cardiovascular risk factors which severely increases the risk of type II diabetes [1] and cardiovascular disease (CVD) [2, 3]. CVD and diabetes are major causes of disability, whose prevalence is increasing worldwide [4]. Therefore, strategies to decrease the onset and progression of MetS and their associated pathologies are of extreme interest.

In terms of the relationship between nutrition and MetS, undernutrition and overnutrition from an energy balance focus are the most studied topics, because a triggering factor of MetS is obesity. Nevertheless, intense research from the last decades has shown that not only energy balance but macro- and micronutrient composition of the diet may influence MetS onset and progression. On those grounds, diet is considered a major environmental insult contributing to the increase in metabolic disease incidence, especially in younger individuals, and current evidence highlights that the overall quality diets such as the Mediterranean diet, the Nordic diet, and Dietary Approaches to Stop Hypertension (DASH) diets protect against MetS or are even able to improve the MetS phenotype [5–7]. A diet is a complex mixture of food and compounds with multiple interactions and varying bioavailability, which hampers the identification and isolation of the effect of one single component in a total meal or diet. Although individual dietary components have to be considered in the context of a whole dietary pattern to evaluate their effects on MetS, phytochemicals such as polyphenols and their metabolites have been shown to modulate MetS through different mechanisms.

Polyphenols are biomolecules found in products from plant origin and have been shown to exert antioxidant and anti-inflammatory effects both *in vitro* and *in vivo* [8]. However, controlled trials in MetS subjects with single phenolic compounds or specific food/beverage/extract do not provide strong evidence for the promising protective effects of polyphenols on cardiovascular diseases as reported in numerous animal and cell studies. This can be attributable to the peculiar characteristics of MetS patients, the use of overdoses in experimental models compared to human trials, and the low bioavailability of polyphenols in the small intestine (5 to 10%).

Therefore, the aim of this review is to update the knowledge on the effects of polyphenol intake in MetS patients, derived from interventional human studies with polyphenols as polyphenol-rich foods and dietary patterns rich in polyphenols.

## 2. Phenolic Compounds: Occurrence, Intake, and Bioavailability

Polyphenols (phenolic compounds) are the most widely distributed secondary metabolites from plants in dietary sources. Although they are not considered essential micronutrients, a huge body of literature evinces their beneficial effects on human health, especially in diets associated with high consumption of fruits and vegetables.

**Figure 1:**
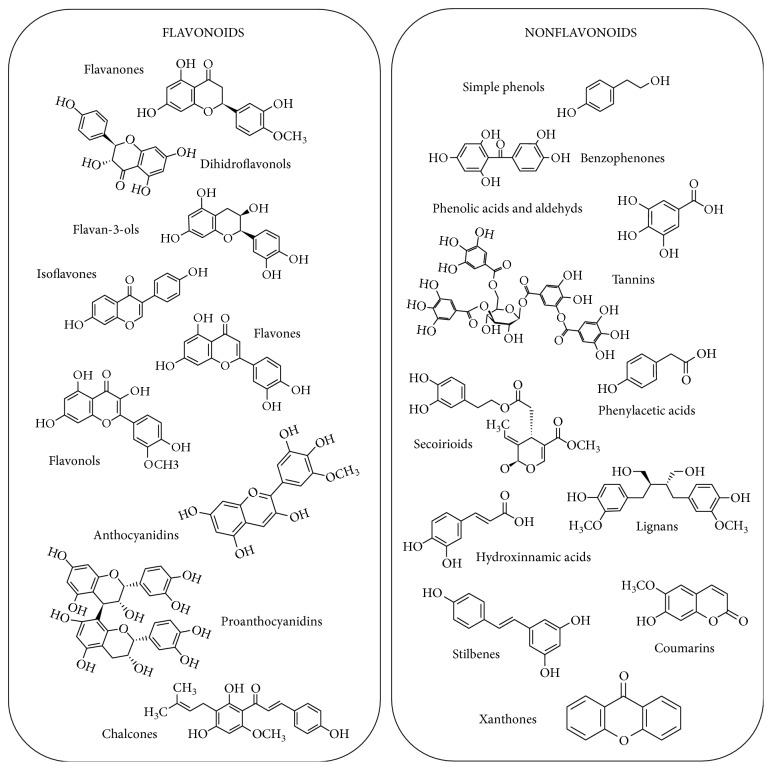
Representative chemical structures of major groups of polyphenols. Adapted from Phenol-Explorer (http://phenol-explorer.eu/) and Andres-Lacueva et al. [132].

Polyphenols share a phenol carbon ring but have different structures, and more than 500 different molecules have been found in foods [9]. According to their chemical structure, they can be divided into two major groups: flavonoids and nonflavonoids, with subsequent subgroups as depicted in Figure 1.  Table 1 displays the main sources of polyphenols in the diet.

Worldwide consumption of polyphenols differs between countries. For instance, mean polyphenol intake of an adult subject is about 283–1000 mg of total polyphenols/day in France [10, 11], 500–1100 mg/day in Spain [12], about 700 mg/d in Italy [13, 14], 890 mg/day in Finland [15], 534 mg/day in Brazil [16], and around 1500 mg/day in Japan [17], while total flavonoid intake is about 190 mg/day in the UK and Ireland [18], about 240–350 mg/day in the US [19, 20], around 450 mg/day in Australia [21], between 50 and 500 mg/day in China [22], and around 320 mg/day in Korea [23]. In addition, there is a great variability in polyphenol intake within countries depending on the type of diet consumed. Because polyphenols are from plant origin, vegetarians and vegans should have higher intakes of polyphenols than people who follow westernized diets. However, in the US and Canada, coffee consumption is more determinant in the amount of total polyphenol intake than the dietary pattern itself [24].

Besides differences between countries and dietary patterns, differences in polyphenol intake can also be attributed to the analytical method used to quantify polyphenols in foods. To determine polyphenol content in foods, there are two main approaches: by measuring total polyphenol content with the Folin-Ciocalteu colorimetric assay (by quantifying the total reducing capacity of a sample) and by measuring single polyphenols or metabolites by chromatographic techniques [25]. The Folin-Ciocalteu method gives higher values of polyphenols than chromatographic techniques because several other reducing agents contained in foods such as vitamin C or some nitrogen-containing compounds [26] interfere in the quantification. Although it is an estimation of total polyphenols, it should strictly be considered a measurement of the *in vitro* antioxidant capacity of foods. On the other hand, chromatographic techniques offer higher sensitivity and sensibility than the colorimetric methods.

To determine polyphenol intake in humans, there are also two different approaches: by determining biomarkers of intake (namely, total polyphenols or single polyphenols or their metabolites in biological samples with chromatographic techniques or again with the Folin-Ciocalteu method) or by estimating its intake.

The estimation of the intake of polyphenols is performed by administering food frequency questionnaires or food recalls to the study subjects and translating this information to single or total polyphenols by using databases to calculate polyphenol intake from consumed food. The major public databases used for polyphenol content of foods and beverages are the United States Department of Agriculture (USDA) databases for flavonoids, proanthocyanidins, and isoflavones and the Phenol-Explorer database. USDA databases only present values for flavonoids, but Phenol-Explorer is a more complete database because it gathers information on all classes and types of polyphenols, including their metabolites [27]. Nevertheless, this approach is limited due to self-reporting bias and because of the seasonal and geographical variability in the polyphenolic composition of foods, the ripeness of the food at time of harvest and its storage before consumption, and the limited data on the polyphenolic composition of food databases. Moreover, polyphenols are not homogeneously distributed within foods but are usually concentrated in the outer layers. Therefore, peeling or processing foods may substantially decrease its polyphenol content [28]. In addition, cooking foods provokes either losses or increases in some polyphenol content of foods [29].

The use of the Folin-Ciocalteu method carries the same limitations described above, and thus, chromatographic analyses provide the most reliable results, although only few polyphenols and their metabolites have been quantified in fluids or tissues. In addition, given the low bioavailability of polyphenols (5–10%) [30] and that maximal plasma concentrations are reached within the first two hours after ingestion and fall to baseline levels within 8 to 12 hours [31], 24 h urine provides a more accurate measure of the total polyphenol absorption, metabolism, and excretion over a 24 h period, even for polyphenols with short half-lives [32].

## 3. The Metabolic Syndrome

MetS is a cluster of several interrelated and well-documented cardiovascular risk factors (hyperglycemia, hypertension, dyslipidemia, insulin resistance, and central adiposity) that may provably result from the increasing prevalence of obesity and seems to be triggered by insulin resistance [33]. Many international organizations and expert groups, such as the World Health Organization (WHO), the European Group for the Study of Insulin Resistance (EGIR), the National Cholesterol Education Program Adult Treatment Panel III (NCEP-ATPIII), the American Association of Clinical Endocrinology (AACE), the International Diabetes Federation (IDF), and the American Heart Association/National Heart, Lung, and Blood Institute (AHA/NHLBI), have attempted to incorporate all the different parameters used to define MetS [34, 35]. However, the consensus definition of the IDF [34] seems the most suitable for practical use in clinical medicine, taking into account the incorporation of different thresholds for different ethnicities as shown in Table 2, recognizing that the risk associated with a particular waist measurement will differ in different populations.

**Figure 2:**
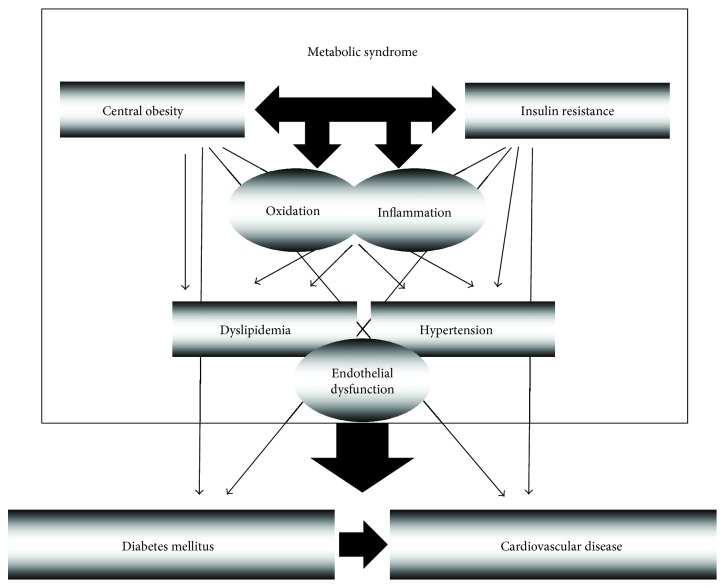
Pathological processes involved in the metabolic syndrome and their potential interactions.

As depicted in Figure 2(), MetS is a pathological condition defined by a chronic, systemic, and low-grade inflammation [36] and oxidative status and characterized by the cluster of three or more independent CV risk factors, namely, abdominal obesity, hyperglycemia/insulin resistance, hypertriglyceridemia, low high-density lipoprotein (HDL) and/or high low-density lipoprotein (LDL) cholesterol, and high blood pressure [37], in which each factor contributes to the development and progression of each other. Overall, this leads to excessive tissue damage, endothelial dysfunction, thrombosis, insulin resistance, and high blood pressure. Accumulation of these cardiometabolic risk factors has been associated with increased CVD [38], diabetes, some forms of cancer, arthritis, neurodegenerative diseases [39], and all-cause mortality [40]. The prevalence of both obesity and type 2 diabetes has increased dramatically in recent decades worldwide, and both conditions represent substantial risk factors for the development of atherosclerotic disease and the resulting increased incidence of myocardial infarction and stroke [41].

Worldwide prevalence of MetS ranges between 10 and 84% depending on the ethnicity, age, gender, and race of the population [3, 42, 43]. As an average, one-quarter of the world's population has MetS [44]. Therefore, MetS has been highlighted as a major socioeconomic problem throughout the world.

## 4. Effects of Polyphenol Intake on the Metabolic Syndrome

Considering that a pro-oxidant status and low-grade chronic inflammation are hallmarks of MetS and that its severity seems to depend on the prevalent number of components of MetS, polyphenols appear as good dietary candidates to prevent MetS progression given their well-described antioxidant and anti-inflammatory actions [45]. Furthermore, polyphenols have been shown to improve insulin resistance [46], to decrease blood pressure [47] and body weight [48], and to improve the lipid profile [49]. Nevertheless, dietary strategies may be less effective for patients with a cluster of risk factors of MetS as a whole than for those patients with one or two risk factors. Along this line, the effects of polyphenol intake on healthy volunteers or low-moderate CV risk patients may differ from those on patients with MetS because of their pathological characteristics. Thus, the results reported in humans are still inconsistent, and the metabolic benefits of polyphenols may strongly depend on the population studied. For instance, urolithins are the microbial metabolites of ellagitannin. Ellagitannin-metabolizing phenotypes (urolithin metabotypes A, B, and 0) differ among individuals depending on their body mass index. In fact, urolithin A in MetS patients only correlated inversely with glucose after intake of 30 g of nuts, while in healthy and overweight patients, urolithin A positively correlated with apolipoprotein A-I and HDL cholesterol after the intake of nuts [50]. Along the same line, a systematic review on the effects of grape polyphenols on MetS components [51] shows differential effects of grape polyphenols on MetS components according to the number of components in each patient. Moreover, in healthy volunteers, extra virgin olive oil consumption improved glycemia, insulin sensitivity, and the inflammatory phenotype, but these effects were observed in a significantly lesser extent in MetS patients [52]. A recent systematic review also postulates that polyphenols are effective in reducing some MetS features, but there is no single food, extract, or polyphenol able to act on all MetS features [53], suggesting that the whole dietary pattern and not food or extract supplements may contribute in controlling MetS progression. Considering their low bioavailability and metabolism, the protective functions of polyphenols may become effective only through frequent and sustained intake at a long term, in the context of a healthy and diversified diet. Given the complex nature of MetS, we aimed to review the modulation of MetS features by polyphenol intake with a special focus on MetS patients, defined according to the IDF, beyond their anti-inflammatory and antioxidant effects.

### 4.1. Epidemiological Evidence

#### 4.1.1. Association between Polyphenol Intake and Central Obesity

In the past decades, polyphenols have attracted media interest because of their potential effects on lipid and energy metabolism and on reducing body weight. Several *in vitro* and animal studies have proposed different mechanisms by which polyphenols could play a potential role in reducing obesity, but few cohort studies have evaluated the association between polyphenol intake and body weight. In addition, interventional trials are very limited too.

In a Chinese cohort, regular tea consumers showed less percentage of body fat and waist-to-hip ratio compared to subjects who did not consume tea on a regular basis [54]. A longitudinal analysis from the Netherlands Cohort Study has shown that increased intake of flavones, flavonols, and catechins is associated with a lower increased body mass index (BMI) associated with age in women but not in men [55]. Chocolate intake in a cohort of subjects under statin treatment without known cardiovascular disease or diabetes was associated with lower BMI, despite that higher chocolate intake was associated with higher calorie and saturated fat intake [56]. However, as recently reviewed, resveratrol intake does not mediate body weight loss in overweight/obese patients, showing a great difference in the response to resveratrol intake between animal and human studies [57].

Despite the potential effects of polyphenols on reducing body weight, a recent systematic review points out that the weight loss induced by polyphenols is not clinically relevant in overweight and obese individuals [58]. In addition, many of the interventional trials have a duration of less than 3 months, and therefore, long-term randomized interventional trials are needed to properly elucidate the role of polyphenols in weight loss and obesity prevention.

#### 4.1.2. Association between Polyphenol Intake and Insulin Resistance

Polyphenol intake has been related to decreased risk of insulin resistance and type 2 diabetes. In a Mediterranean cohort at high cardiovascular risk, total polyphenol, total flavonoid (flavanones and dihydroflavonols), and stilbene intake was associated with decreased risk of type 2 diabetes [12]. In the Nurses' Health Studies (NHS) I and II, urinary excretion of flavanones (naringenin and hesperetin) and flavonols (quercetin and isorhamnetin), as well as caffeic acid, was associated with about 39%–48% decreased risk of type 2 diabetes at a middle term (5 years) but not at a long term (up to 11 years) [59]. In this study, only hesperetin was associated with decreased long-term risk of type 2 diabetes. However, in another substudy of women from the NHS I and II and men from the Health Professionals Follow-up Study, higher intakes of anthocyanins, but not of total flavonoids or other flavonoid subclasses, were significantly associated with lower risk of type 2 diabetes [60]. Oppositely, the European Prospective Investigation into Cancer and Nutrition- (EPIC-) InterAct case-cohort study observed that flavan-3-ol monomers and proanthocyanidin dimers and trimers, but not proanthocyanidins with greater polymerization degrees, were associated with decreased risk of type 2 diabetes [61]. Nevertheless, the Iowa Women's Health Study found no association between flavonoid consumption and the risk of type 2 diabetes [62], in accordance with the Women's Health Study [63], in which dietary intake of total flavonols and flavones, quercetin, kaempferol, myricetin, apigenin, and luteolin was not associated with decreased risk of type 2 diabetes. However, apple and tea consumption was associated with lower risk of type 2 diabetes. In this line, green tea (but not black tea) intake of more than 3 cups/day has been inversely associated with type 2 diabetes risk in a Japanese cohort [64]. On the other hand, the relationship between coffee intake and type 2 diabetes is a matter of controversy [65].

#### 4.1.3. Association between Polyphenol Intake and Dyslipidemia

A meta-analysis has shown that green tea but not black tea [66] consumption decreases total and LDL cholesterol [67] with no effects on HDL cholesterol, although some studies observed that green tea consumption increased HDL cholesterol levels [68]. In the TOSCA.IT study with type 2 diabetic patients, high polyphenol intake is associated with slightly lower levels of LDL and triglycerides and higher levels of HDL cholesterol [14]. In the Moli-sani cohort, higher intakes of polyphenols were associated with lower total and LDL cholesterol, lower triglycerides, and higher levels of HDL cholesterol [69]. In the PREDIMED cohort, increased polyphenol intake at 5 years measured by total urinary polyphenol excretion was inversely associated with triglyceride levels but not with total, LDL, or HDL cholesterol levels [70]. In a subset of the NHANES cohort, urinary enterolignan concentrations (enterolactone and enterodiol) were associated with increased levels of HDL cholesterol and decreased levels of triglycerides, but no association was found for total or HDL cholesterol [71]. In a subset cohort of the ATHENA study, total polyphenol and anthocyanin intakes were not associated with an improved lipid profile, but individuals with the serum paraoxonase/arylesterase 1 single-nucleotide polymorphisms rs854549 and rs854552 showed a positive association between HDL cholesterol levels and total polyphenol and anthocyanin intakes [72].

#### 4.1.4. Association between Polyphenol Intake and Hypertension

Several observational studies have revealed a positive correlation between greater intake of fruits and vegetables and a decreased prevalence of hypertension (reviewed in [73]). In a Mediterranean population at high cardiovascular risk, total polyphenol intake measured by urinary polyphenol excretion was associated with lower blood pressure levels and lower prevalence of hypertension [74]. However, in a Brazilian cohort, an inverse relationship was found for hypertension and intake of lignans, stilbenes, tyrosols, alkylphenols, and other polyphenols, but not for total polyphenol, flavonoid, or phenolic acid intake [75]. A meta-analysis of the effects of hibiscus tea showed that supplementation with this sour tea decreased blood pressure levels [76], and another meta-analysis has shown that green tea consumption decreases systolic blood pressure, although the size of the effects appears to be relatively small (about 2 mmHg) [67] in accordance with another meta-analysis [77]. High intake of blueberries and strawberries (rich in anthocyanins) has been associated with decreased risk of hypertension in subsets of the cohorts of women from the NHS I and II and men from the Health Professionals Follow-up Study (HPFS) [78]. Furthermore, in a cross-sectional study on women aged 18–75 years old, higher anthocyanin intake, but not intake of other flavonoids, was associated with lower systolic blood pressure and mean arterial pressure, whereas anthocyanin and flavone intake was associated with lower pulse wave velocity [79]. However, a recent meta-analysis pinpoints that blueberry supplementation (and consequently anthocyanin supplementation) does not decrease blood pressure [80]. Therefore polyphenols, and specifically flavonoids, show potential antihypertensive effects, which may differ in relation to the disease status (healthy versus pre-hypertensive versus hypertensive individuals).

#### 4.1.5. Association between Polyphenol Intake and the Metabolic Syndrome

Several epidemiological studies have observed a negative association between polyphenol intake and MetS rates. In a cross-sectional study comprising more than 8800 subjects, BMI, waist circumference, blood pressure, and triglycerides were significantly lower among individuals in the higher quartiles of polyphenol intake, assessed by food frequency questionnaires. In addition, the odds ratio for MetS was about 0.75 for subjects at the highest quartile of polyphenol intake [81].

Coffee consumption has been inversely associated with the prevalence of MetS in Korean women [82] and Japanese [83] and Danish men and women [84], although these associations may vary depending on the body mass index [85].

Tea drinking, especially 240 ml or more of tea daily, was inversely associated with incidence of MetS in elderly Taiwanese males [86]. A cross-sectional study of US adults showed that intake of hot (brewed) tea, but not of iced tea, was inversely associated with obesity and biomarkers of MetS and CVDs [87]. On the other hand, a cross-sectional study of a Japanese cohort observed no such association [83].

In a Spanish cohort, moderate red wine consumption was negatively associated with MetS prevalence, accompanied by reduced waist circumference, blood pressure, and fasting glucose and higher HDL levels [88]. On the other hand, in another Spanish cohort, no relationship was found between wine consumption and MetS prevalence, but beer consumption was associated with higher risk for MS [89].

Overall, epidemiologic data is very useful from a hypothesis-generating perspective, but usually, nutritional data relies on the transformation of food frequency questionnaires to food or single polyphenol intake, and large epidemiological trials do not often use reliable biomarkers of intake. Therefore, caution should be taken when interpreting epidemiological data.

### 4.2. Evidences from Interventional Studies

As previously stated, MetS is triggered by a maintained pro-oxidant status and low-grade chronic inflammation. Therefore, antioxidant and anti-inflammatory compounds may appear protective for the MetS onset and progression, but despite the well-known antioxidant effects of polyphenols *in vitro* in numerous clinical studies, antioxidant and anti-inflammatory effects were not significant after polyphenol supplementation in patients with MetS (reviewed in [53]).

#### 4.2.1. Antioxidant Activity of Polyphenols

In MetS women, 480 mL/day of cranberry juice for 8 weeks increased plasma antioxidant capacity and decreased oxidized LDL and malondialdehyde [90], and 50 g freeze-dried blueberries for eight weeks decreased serum oxidized LDL and malondialdehyde and increased hydroxynonenal concentrations in both men and women with MetS [91]. However, in women with MetS, 22 g freeze-dried blueberry powder for eight weeks showed no effect on superoxide dismutase levels [92], but 300 mL/day of pomegranate juice for 6 weeks decreased the levels of thiobarbituric acid reactive substances (TBARS) in erythrocytes [93]. MetS patients receiving 10 mL/day of extra virgin olive oil showed increased total radical-trapping antioxidant parameter (TRAP)/uric acid ratio, but no effects were observed on the levels of hydroperoxide, advanced oxidation protein product (AOPP) or AOPP/TRAP, and TRAP/AOPP indexes [94].

In MetS patients, a Mediterranean diet for five years supplemented with extra virgin olive oil or nuts increased plasma levels and activity of superoxide dismutase and catalase, increased levels of nitrates, and decreased xanthine oxidase activity [95], and a Mediterranean-style diet for 3 months decreased lipid and protein oxidation and increased plasma, erythrocyte, and platelet antioxidant enzymes [96].

The antioxidant potential of polyphenols is defined by their chemical structure, the number and position of hydroxyl groups, conjugation groups, degree of glycosylation, and the presence of donor electrons in the ring structure, considering that the aromatic group is able to endure the disappearance of electrons. However, whether the effects of polyphenols are relevant in oxidative stress remains to be elucidated. Despite their low bioavailability and rapid metabolism and elimination, the antioxidant effects of polyphenols may be of potential clinical relevance when considered in the context of a diet rich in fruits and vegetables. However, being polyphenol-rich foods and also antioxidant vitamin-rich foods, the antioxidant activity observed in these trials may be a sum of the effects of antioxidant vitamins and polyphenols, because polyphenols represent <1-2% of the plasma antioxidants, which include proteins, ascorbate, tocopherol, carotenoids, bilirubin, uric acid, and several other compounds [97]. Therefore, polyphenols may exert their protective effects far beyond their antioxidant activity.

#### 4.2.2. Anti-Inflammatory Activity of Polyphenols

Besides the antioxidant activity of polyphenols, they exert effects on enzymes, cell signaling pathways, and gene expression related to inflammation that may better explain their beneficial effects on endothelial function, metabolic disturbances, and vascular inflammation. Berry supplementation in the form of juice, powder, or extract has been shown to decrease serum levels of interleukin- (IL-) 12, monocyte expression of monocyte-to-macrophage differentiation-associated (MMD) and C-C motif chemokine receptor 2 (CCR2), and the overall inflammation score [98] and to improve endothelial function measured by the reactive hyperemia index [99] in MetS patients. However, in those patients, berry supplementation had no effect on C-reactive protein (CRP) [91, 92], intercellular adhesion molecule-1 (ICAM-1), vascular adhesion molecule-1 (VCAM-1), adiponectin [91], IL-6 [90], CRP, tumor necrosis factor- (TNF-) *α*, or monocyte chemoprotein- (MCP-) 1 [100]. On the other hand, men with MetS show decreased ICAM-1 levels and increased flow-mediated dilation after 46 g/day of freeze-dried grape polyphenol powder for 30 days [101], and green tea supplementation for eight weeks through infusion or powder did not significantly modify serum levels of adiponectin, CRP, IL-6, IL-1*β*, VCAM-1, ICAM-1, leptin, or leptin : adiponectin ratio [102] in both men and women with MetS. In those patients, a Mediterranean-style diet for 3 months decreased CRP levels [96] and serum concentrations of IL-6, IL-7, and IL-18 and increased the endothelial function score after 2 years [103]. These controversial results may partly be explained by differences in polyphenol doses in each trial and also suggest that MetS should be addressed through a multifactorial approach considering the whole dietary pattern and also increased physical activity, known to be a good anti-inflammatory strategy [104].

#### 4.2.3. Acute Interventions with Polyphenol-Rich Foods in Patients with Metabolic Syndrome

As pinpointed in a previous section, given the low bioavailability of polyphenols and that maximal plasma concentrations are reached within the first two hours after ingestion and fall to baseline levels within 8 to 12 hours, the protective effects of polyphenols may be reached through a long-term regular, daily basis consumption. However, minimizing postprandial metabolic stress may help in the control of the chronic progression of MetS. Therefore, polyphenols may be more active when consumed in the principal meals, which provokes greater postprandial metabolic stress. While several studies reported beneficial postprandial effects of polyphenol intake on healthy subjects or on subjects with one or two cardiovascular risk factors, very few acute interventional trials have been performed in MetS patients. Diets rich in polyphenols have been shown to decrease postprandial triglyceride total area under the curve in plasma and large VLDLs, as well as to decrease 24 h urinary 8-isoprostane in these patients [105]. Acute intake of extra virgin olive oil improved glycemia and insulin sensitivity in healthy subjects but not in MetS patients. In addition, the authors observed that acute extra virgin olive oil consumption switched peripheral blood mononuclear cells to a less deleterious inflammatory phenotype, but weaker effects were observed in patients with MetS [52]. Taking into account these differences and given the fact that MetS could affect polyphenol bioavailability as shown in rats [106], more acute interventional trials in MetS patients are warranted in order to elucidate the capability of polyphenols to minimize postprandial metabolic stress in MetS patients.

#### 4.2.4. Long-Term Interventions with Polyphenol-Rich Foods in Patients with Metabolic Syndrome

On the other hand, clinical trials are currently the best approach to demonstrate the effects of foods or food compounds such as polyphenols on human health. Nevertheless, it has to be taken into account that the fact that a food or a single polyphenol modulates some or several biomarkers related to MetS may not imply an improvement of the MetS progression itself. In addition, while several randomized trials have evaluated the effects of polyphenols on patients with abdominal obesity and or some of the MetS components, few randomized trials have evaluated the effects of polyphenols on MetS patients. Therefore, more and large randomized trials are needed to definitively elucidate the role of polyphenols on MetS. In this subsection and in Table 3, the main clinical trials on polyphenols and MetS are summarized.

*(1) Fruit Polyphenols*. Berries are very rich in anthocyanins, being pelargonidin, cyanidin, delphinidin, petunidin, peonidin, and malvidin the most predominant. Randomized trials analyzing the effect of berry consumption (as fresh fruit, juice, or extracts) are performed at a relatively short term, and therefore, their long-term effects still remain unknown. Blueberry consumption has been shown to decrease blood pressure [92] and oxidative stress [91] and improve endothelial function [99] and insulin sensitivity [100] in MetS patients. On the other hand, inconsistent results have been found for other berries in the improvement of MetS. In MetS patients, neither bilberry [98] nor cranberry supplementation [90] showed differences in body weight, glucose, or lipid metabolism, compared to a control group. However, compelling evidence shows that overall berry intake improves the oxidative and inflammatory profile, as reviewed [107].

In middle-aged women with metabolic syndrome, 300 mL of pomegranate juice (rich in flavonoids) daily for 6 weeks decreased erythrocyte but not plasma TBARS levels. Nevertheless, they observed no effects on blood pressure [93].

*(2) Olive Oil Polyphenols*. It is worth pointing out that olive oil polyphenols are contained in extra virgin olive oil, whereas olive oil is almost deprived of polyphenols because of the refining process [108]. Polyphenols from extra virgin olive oil, mainly (hydroxy) tyrosol and oleuropein, elicit antioxidant and anti-inflammatory effects, improve the lipid profile and the endothelial function, and may exert some antithrombotic effects [109]. Although the beneficial effects of olive oil polyphenols on the general population are not under debate, to our knowledge, only a clinical trial has been performed to evaluate the effects of extra virgin olive oil as a single food in MetS patients. These patients showed decreased waist perimeter after consuming 10 mL of extra virgin olive oil a day for 3 months, but no changes in the lipid profile, glucose, insulin resistance, or blood pressure were observed [94].

*(3) Tea Polyphenols*. Tea is a beverage especially rich in catechins. The beneficial effects of tea (higher for green than for black tea intake) on MetS have generally been observed in most human studies at least at three to four cups or more per day. One of the protective main effects of tea consumption in MetS is by reducing body weight [110], by decreasing digestion and absorption of some macronutrients, by altering the gut microbiota, by inhibiting anabolism, and by stimulating catabolism in liver, muscle, and adipose tissues [48]. However, current evidence suggests that this effect may only be observed at a short term and not at a long term [111].

*(4) Grape and Red Wine Polyphenols*. Grape and wine (red wine in a higher degree than white wine) are very rich in phenolic acids, stilbenes, flavonols, dihydroflavonols, anthocyanins, catechins, and proanthocyanidins. Consumption of dried grape polyphenol powder for a month resulted in decreased systolic blood pressure [112] and increased flow-mediated dilation in men with MetS, but no effects were observed in insulin resistance, lipid profile, or body weight [101]. Intake of red wine or dealcoholized red wine for 30 days significantly decreased SBP, DBP, glucose, triglycerides, total cholesterol, and C-reactive protein and increased HDL levels in MetS patients [113]. In parallel and as will be further discussed, red wine polyphenols (in both interventions) increased the number of fecal bifidobacteria and *Lactobacillus* (intestinal barrier protectors) and butyrate-producing bacteria and decreased lipopolysaccharide- (LPS-) producing bacteria, which was translated to lower LPS systemic levels, suggesting that red wine polyphenols are capable of switching microbiota phenotype to a more protective one in patients with MetS [113]. However, a recent meta-analysis concludes that grape polyphenols do not have a significant effect on glycemia, blood pressure, or the lipid profile in MetS patients, although limited evidence suggests a positive effect on insulin sensitivity [51], in contraposition to another meta-analysis that concluded that grape polyphenols are able to decrease systolic but not diastolic blood pressure in MetS patients [114].

*(5) Mediterranean Diet*. The Mediterranean diet is characterized by the high consumption of polyphenol-rich foods such as fruits, vegetables, nuts, olive oil, aromatic plants, spices, whole-grain cereals, and red wine. Several cross-sectional and prospective studies have suggested that the Mediterranean diet has protective effects on different components of MetS [115] and its associated low-grade inflammation and pro-oxidative status. Intervention with a Mediterranean diet for five years supplemented with extra virgin olive oil or nuts increased plasma antioxidant capacity and decreased xanthine oxidase activity in MetS patients [95]. This is in concordance with another study with MetS patients, which reported that a Mediterranean-style diet for 3 months improved the antioxidant enzyme activities in plasma, erythrocytes, and platelets [96]. Moreover, MetS patients under a Mediterranean diet for 2 years showed reduced prevalence of MetS by decreasing body weight, reducing serum concentrations of inflammatory markers, decreasing insulin resistance, and improving endothelial function [103]. Concurrently, Mediterranean diet intervention for one year supplemented with extra virgin olive oil or nuts was associated with decreased incidence [116] and increased reversion of MetS [117], even at five years of intervention [118], suggesting that the Mediterranean diet may be a promising tool for both MetS prevention and management. In the same cohort, one-year intervention with a Mediterranean diet supplemented with extra virgin olive oil or nuts has been shown to decrease blood pressure, and these changes were associated with a significant increase in total polyphenols in urine and plasma nitric oxide [119]. Overall, a Mediterranean diet for five years supplemented with extra virgin olive oil or nuts, despite the increased fat intake, was not associated with increased visceral adiposity or body weight [120], suggesting that indeed, olive oil and nut polyphenols, among other compounds, may have an effect on body weight maintenance or loss.

### 4.3. Gut Microbiota and Metabolic Syndrome

The human colonic microbiota is a large and complex microbial community. In total, over 1000 bacterial species have been identified, of which many remain uncultured, with about 160 species being found in the gut of any individual, making an important contribution to human metabolism and health by contributing enzymes that are not encoded by the human genome [121]. Given the low bioavailability of polyphenols, the remaining polyphenols (90–95% of total polyphenol intake) may accumulate in the large intestinal lumen up to the millimolar range where they are subjected to the enzymatic activities of the gut microbial community prior to their reabsorption, modulating the composition of the microbiota and influencing the metabolic balance and the health status. In this line, red wine intake has been shown to increase global fecal microbial diversity in healthy volunteers [122], and different profiles of urinary polyphenol metabolites derived from microbial metabolism have been shown after one-month red wine intake between healthy individuals and obese/diabetic patients [123].

Once the polyphenols have been metabolized to their aglycones or the polymers have been converted to monomers, they are extensively degraded by components of the colonic microbiota via dehydroxylation, decarboxylation, and ring breakage ultimately generating simpler phenolic compounds, such as hydroxyphenyl-acetic acids and hydroxyphenylpropionic acids [124]. Given the high variability in the microbial composition between individuals, polyphenols may be differently metabolized, thus affecting the bioavailability of both the parental polyphenols and their metabolites and influencing their bioactivity. For instance and as previously mentioned, daidzein (a soy isoflavone) is metabolized by two different pathways depending on the gut microbiota of the subjects, producing O-desmethylangolensin by *Clostridium*, or on (S)-equol via dihydrodaidzein and tetrahydrodaidzein by *Streptococcus intermedius*, *B. ovatus*, *Ruminococcus productus*, *Eggerthella sp.* Julong732, *Adlercreutzia equolifaciens*, *Slakia isoflavoniconvertens*, and *Slakia equolifaciens* [125]. In addition, different responses to a mix of epigallocatechin-3-gallate and resveratrol have been observed between men and women. In men, this polyphenol mixture decreased the amount of fecal bacteroidetes and tended to decrease *Faecalibacterium prausnitzii*, but these changes were not observed in women [126], highlighting the complex nature of intestinal microbiota. Moreover, MetS features may also influence intestinal microbiota and polyphenol metabolism and therefore their bioactivity [127, 128]. A randomized study in MetS patients has shown that bioavailability of ellagitannins depends on the gut composition of microbiota [98]. As previously remarked, ellagitannin-metabolizing phenotypes (urolithin metabotypes A, B, and 0) differ among individuals depending on their body mass index, and urolithin A in MetS patients only correlated inversely with glucose after intake of 30 g of nuts, while in healthy and overweight patients, urolithin A positively correlated with apolipoprotein A-I and HDL cholesterol after the intake of nuts [50]. In MetS patients, red wine polyphenols in the form of red wine or dealcoholized red wine significantly increased the number of fecal protective species (bifidobacteria and *Lactobacillus*) and butyrate-producing bacteria (*Faecalibacterium prausnitzii* and *Roseburia*), consequently decreasing the amount of groups of nonprotective bacteria such as LPS producers (*Escherichia coli* and *Enterobacter cloacae*) and approaching the microbiome profile of MetS patients to the profile of healthy volunteers. Indeed, before the red wine intervention, MetS patients had higher amounts of Proteobacteria and Firmicutes compared to healthy subjects, while after the red wine and dealcoholized red wine intake periods, no significant differences were observed between healthy individuals and MetS patients [113]. Overall, the (complex) interaction between nutrients and the gut microbiome is an emerging field of intensive research that will provide new and promising insights into the relationship between nutrition and health in the near future.

## 5. Conclusions

**Figure 3:**
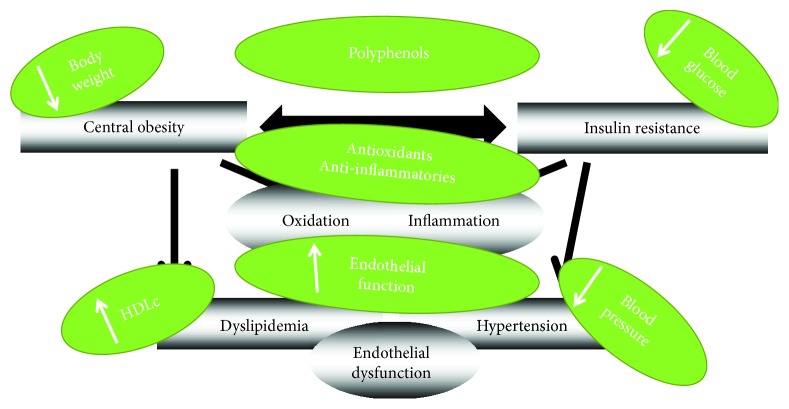
Main potential effects of polyphenols on metabolic syndrome components.

As summarized in Figure 3(), compelling evidence suggests that polyphenols at maintained doses may delay or prevent MetS onset by decreasing body weight, blood pressure, and blood glucose and by improving lipid metabolism. Because of the complex polyphenol composition of polyphenol-rich foods, it is difficult to emphasize the bioactivity of a specific polyphenol. Moreover, it seems plausible that they have additive or synergistic effects. Therefore, high intake of polyphenol-rich foods such as nuts, fruits, vegetables, seasonings with aromatic plants, spices, and virgin olive oil may be the cornerstone of a healthy diet preventing the development and progression of MetS. However, human epidemiological and interventional studies have shown inconsistent results. There is a small number of interventional studies evidencing the benefits of polyphenol intake in the improvement of MetS phenotype in these patients, and more long-term randomized trials are warranted in order to evaluate the possible preventive effects of a higher consumption of polyphenols by a combination of their diverse dietary sources, as suggested by some epidemiological observations [12, 61, 129–131] and in order to conclude whether single polyphenols or polyphenol-rich foods are indeed related to a reduction in MetS-related symptoms or whether their action is merely affecting a biomarker.

In conclusion, current evidence suggests that polyphenol intake has the potential to alleviate MetS components. However, there is still a long way to run before establishing the role of polyphenols in MetS progression. Safe doses have to be determined, as the effects greatly vary among polyphenols and food sources, and no specific food or polyphenol is able to improve all components of MetS. Overall, a varied diet rich in polyphenol-rich foods may be beneficial in the onset and progression of MetS and in decreasing the associated risk of developing diabetes or cardiovascular disease.

## Figures and Tables

**Table 1:** Major food sources of dietary polyphenols.

Food group	Polyphenols
Wine	Phenolic acids, stilbenes, flavonols, dihydroflavonols, anthocyanins, flavanol monomers (catechins), and flavanol polymers (proanthocyanidins)
Beer	Prenylated flavonoids, phenolic acids, simple phenols, flavanols, hydroxycoumarins, flavonols, and flavones
Coffee	Phenolic acids
Tea	Catechins, phenolic acids, flavonols, and proanthocyanidins
Cocoa	Flavanols (catechins and proanthocyanidins), phenolic acids, flavonols, some stilbenes, simple phenols, and isocoumarins
Vegetables	Flavonols (kaempferol and quercetin derivatives) and hydroxycinnamic acids (cabbages) Hydroxycinnamic acids, flavonols, and flavanones (tomatoes) Flavonoids, phenolic acids, and capsaicinoids (pepper) Hydroxycinnamic acids and anthocyanins (eggplant) Hydroxycinnamic acids, flavones, and flavonols (leaf vegetables) Flavonols such as quercetin (onions) Phenolic acids (roots)

Fruits	Anthocyanins, ellagitannins, and proanthocyanidins (berries) Flavanone glycosides, polymethoxylated flavones, and traces of flavonols and hydroxycinnamic acids (citrus) Chlorogenic acids, anthocyanins, flavonols, catechins, and proanthocyanidins (pommes and drupes)
Nuts	Catechins, proanthocyanidins, ellagitannins, and ellagic acid
Pulses	Proanthocyanidins, flavonols, flavanones, and hydroxycinnamic acids
Soy	Isoflavonoids
Virgin or extra virgin olive oil	Tyrosols
Sesame oil	Lignans and phenolic acids
Aromatic plants	Phenolic acids, flavones, phenolic diterpenes, and flavanones
Spices	Phenylpropenes, phenolic acids, flavones, and flavonols

Data extracted from Phenol-Explorer (http://phenol-explorer.eu/).

**Table 2:** Metabolic syndrome definition.

Cardiovascular risk factor	Measurement	Definition		
Central obesity	Waist circumference	Region	Men	Women
		Asia	≥90 cm	≥80 cm
		Europe, Africa, and Middle-East	≥94 cm	≥80 cm
		America	≥102 cm	≥88 cm
Insulin resistance	Fasting blood glucose	≥100 mg/dL (5.55 mmol/L)		
Hypertension	Blood pressure	≥130/85 mmHg		
Dyslipidemia^∗^	Fasting blood triglycerides	≥150 mg/dL		
	Fasting blood HDL cholesterol	≤50 and ≤40 mg/dL for women and men, respectively		

According to the International Diabetes Federation (IDF), metabolic syndrome is defined as central obesity plus 2 or more cardiovascular risk factors [133], and according to the American Heart Association (AHA), metabolic syndrome is defined by having 3 or more cardiovascular risk factors [134]. ^∗^High triglyceride levels and low HDL cholesterol levels are considered independent risk factors for the definition of metabolic syndrome. HDL denotes high-density lipoprotein.

**Table 3:** Summary of interventional trials with polyphenol-rich foods on metabolic syndrome.

Ref.	Type of study	Number of patients	Patients' characteristics	Age (years)	Intervention	Dose	Duration	Measured outcomes	Results
[92]	Placebo-controlled	48	Postmenopausal women with pre- and stage 1 hypertension	55–65	Blueberries	22 g/day powder	8 weeks	Blood pressure, arterial stiffness, CRP, nitric oxide, and superoxide dismutase	Decreased blood pressure and arterial stiffness and increased nitric oxide after blueberry intervention: no effects on CRP
[91]	Placebo-controlled	48	MetS	47–53	Blueberries	50 g/day powder	8 weeks	Blood pressure, lipid profile, HOMA index, oxidation, and inflammation parameters	Decreased blood pressure, no changes in body weight, HOMA index or lipid profile. Decreased oxLDL, MDA, and HNE. No changes in inflammatory biomarkers
[99]	Placebo-controlled	44	MetS	53–61	Blueberries	45 g/day powder	6 weeks	Blood pressure, endothelial function, and insulin sensitivity	Improved endothelial function. No changes in blood pressure or insulin sensitivity
[100]	Placebo-controlled	32	Obese, nondiabetic, and insulin-resistant	46–57	Blueberries	45 g/day powder	6 weeks	Insulin sensitivity, inflammatory biomarkers, and adiposity	Improved insulin sensitivity but no changes in adiposity or inflammatory biomarkers
[98]	Placebo-controlled	27	MetS	43–59	Bilberries	400 g fresh	8 weeks	Body weight, blood pressure, glucose, lipid profile, and inflammatory parameters	Decreased CRP, IL-6, IL-12, and LPS concentrations and decreased expression of MMD and CCR2 in monocytes. No changes in body weight, blood pressure, glucose, or lipid metabolism
[90]	Placebo-controlled	31	MetS women	46–60	Cranberries	480 mL/day juice	8 weeks	Blood pressure, glucose and lipid profile, markers of inflammation, and oxidation	Increased plasma antioxidant capacity and decreased oxLDL and MDA. No changes in blood pressure, glucose and lipid profiles, CRP, and IL-6
[93]	Placebo-controlled	23	MetS women	40–60	Pomegranate	300 mL/day juice	6 weeks	Lipid peroxidation and phospholipid fatty acid composition of plasma and erythrocytes, blood pressure, and lipid profile	Decreased plasma arachidonic acid and increased saturated fatty acids. Decreased TBARS and arachidonic acid and increased monounsaturated fatty acids in erythrocytes. No changes in blood pressure or lipid profile
[94]	Placebo-controlled	102	MetS	43–60	Extra virgin olive oil	10 mL/day	90 days	Blood pressure, BMI, HOMA index, lipid profile, CRP, and oxidative parameters	Decreased waist perimeter and increased TRAP but no changes in blood pressure, lipid profile, HOMA index, BMI, or CRP
[112]	Placebo-controlled	65	Normal and overweight	18–50	Green tea extract	9 capsules/day containing >0.06 g EGCG and 0.03–0.05 g caffeine per capsule	12 weeks	Body weight, fat mass index, resting energy expenditure	No differences in body weight, fat mass index, or resting energy expenditure
[113]	Placebo-controlled	27	MetS	25–80	Grape seed extract	300 or 150 mg/day	4 weeks	Serum lipids, blood glucose, and blood pressure	Decreased blood pressure. No changes in glucose or lipid profile. No differences between doses
[101]	Randomized crossover	24	MetS men	30–70	Grape seed powder or placebo	46 g/day (267 mg polyphenols)	30 days	Blood pressure, endothelial function, lipid profile, glucose, and body weight	Decreased systolic blood pressure and ICAM-1 and increased FMD. No differences in diastolic blood pressure, nitric oxide, body weight, glucose, or lipids
[114]	Randomized crossover	10	MetS		Red wine and dealcoholized red wine	272 mL/day	30 days	Body weight, blood pressure, glucose and insulin, lipid profile, CRP, and LPS	Decreased systolic and diastolic blood pressure, glucose, triglycerides, total cholesterol, CRP, and LPS and increased serum levels of HDL cholesterol. No changes in body weight
[95]	Randomized parallel	75	MetS	55–80	Mediterranean diet supplements with extra virgin olive oil or nuts and control diet	—	5 years	Catalase, SOD, myeloperoxidase, and XOX activities and protein levels; protein carbonyl derivatives; and nitrotyrosine, nitrite, and nitrate levels	Increased plasma activity and protein levels of SOD and catalase, increased plasma nitrate levels, and decreased XOX activity in the Mediterranean diets compared to the control diet
[96]	Case-control	36	MetS and healthy individuals	42–64	Mediterranean diet	—	3 months	Insulin resistance and oxidative and inflammatory status	Decreased plasma, erythrocyte, and platelet antioxidant enzymes and a rise in lipid and protein oxidation, plasma CRP, and fibrinogen in MetS patients
[103]	Randomized parallel	180	MetS	37–50	Mediterranean diet and prudent diet	—	2 years	Endothelial function score, lipid profile and glucose, insulin sensitivity, circulating levels of CRP, and IL-6, IL-7, and IL-18	Decreased body weight and insulin resistance, decreased concentrations of CRP, IL-6, IL-7, and IL-18, and improved endothelial function score in the Mediterranean diet group
[102]	Randomized controlled	35	MetS	40–44	Green tea	Green tea (4 cups/day), green tea extract (2 capsules and 4 cups water/day)	8 weeks	Body weight, lipid profile, blood pressure, and inflammatory biomarkers	Decreased total and LDL cholesterol after green tea extracts. No changes in body weight, blood pressure or in serum levels of adiponectin, CRP, IL-6, IL-1*β*, VCAM-1, ICAM-1, leptin, or leptin : adiponectin ratio
[105]	Randomized parallel	86	Overweight/obese individuals with a large waist circumference and any other component of the metabolic syndrome	44–64	Isoenergetic diets with high and low polyphenol content	—	8 weeks	Fasting and postmeal TRLs and 8-isoprostane concentrations	Reduced fasting triglyceride concentrations and large VLDL, reduced postprandial triglyceride total area under the curve in plasma and large VLDLs, and decreased urinary 8-isoprostane after high polyphenol intake
[52]	Case-control	24	Healthy and MetS	27–38	Extra virgin olive oil	50 mL/single dose	Acute	Glycemia, insulin sensitivity, lipid profile, and gene and miRNA expression of peripheral blood mononuclear cells	Improved glycemia and insulin sensitivity in healthy subjects but not in MetS patients. No changes in lipid profile in either population

Ref.: reference number; MetS: metabolic syndrome; HOMA: homeostasis model assessment of *β*-cell function and insulin resistance; BMI: body mass index; CRP: C-reactive protein; LPS: lipopolysaccharide; SOD: superoxide dismutase; XOX: xanthine oxidase; IL: interleukin; TRL: triglyceride-rich lipoprotein; oxLDL: oxidized low-density lipoprotein; VLDL: very low-density lipoprotein; MDA: malondialdehyde; HNE: hydroxynonenal; MMD: monocyte-to-macrophage differentiation-associated; CCR2: C-C motif chemokine receptor 2; TBARS: thiobarbituric acid reactive substances; TRAP: total peroxyl radical-trapping antioxidant potential; ICAM-1: intercellular adhesion molecule-1; VCAM-1: vascular adhesion molecule 1; FMD: flow-mediated dilation.
